# Successful Reimplantation of a Femoral Stem Fracture After Cementless Total Hip Replacement Using the Femoral Window Technique in a Small Dog

**DOI:** 10.3390/ani15091237

**Published:** 2025-04-28

**Authors:** Yoshiyuki Inoue, Kohei Kuroda

**Affiliations:** Faculty of Agriculture, University of Miyazaki, Miyazaki 889-2192, Japan

**Keywords:** total hip replacement, cementless, reimplantation, stem fracture

## Abstract

Total hip replacement (THR) is a common procedure used in veterinary medicine to treat hip joint diseases, particularly in medium- and large-sized dogs. Although cementless techniques have become the standard owing to concerns regarding the aseptic loosening of cemented implants, complications such as stem fractures can still occur. This study reports a case of a 1-year-old, 2.8 kg Toy Poodle that underwent cementless THR using the Zurich mini-cementless hip system to treat Legg–Calvé–Perthes disease. Six months post-surgery, the dog developed a fractured stem, and a second surgery was performed using the “window technique” to replace the broken stem. The procedure involved creating a small window in the femoral cortex to remove the damaged stem and insert a new one. The dog recovered successfully with no further lameness. This case highlights the effectiveness of the window technique for stem replacement in small dogs and emphasizes the importance of optimal stem selection and positioning during the initial THR.

## Simple Summary

A 1-year-old Toy Poodle underwent a cementless total hip replacement for Legg–Calvé–Perthes disease. Six months after surgery, the dog developed lameness and radiography revealed a stem fracture. A second surgery was performed to replace the broken stem using a “window technique”, where a small opening was made in the femur to remove the damaged stem and insert another stem that was one size larger. The surgery was successful, and the dog recovered without further lameness. This case demonstrates that the window technique can be effective for stem replacement in small dogs and emphasizes the importance of the careful placement and selection of the stem during the initial surgery.

## 1. Introduction

In veterinary orthopedics, total hip replacement (THR) is one of the most common salvage treatments for hip joint diseases in which the restoration of joint function is impossible. Cement has been conventionally used to fix the stem and cup; however, at present, the cementless system is preferred to avoid the risk of the aseptic loosening of the implants in the long term. Good results have been reported, mainly in medium and large dogs [1,2,3,4]; in recent years, there have been increasing reports on its use in small dogs [5,6,7]. Common complications associated with cemented and cementless THRs include luxation, acetabular cup displacement, infection, aseptic loosening of the stem or cup, femoral and acetabular fractures, subsidence, and sciatic neurapraxia [8,9,10,11,12,13,14,15,16,17]. Additionally, stem fractures have also been reported as rare complications [18]. There are reports in the veterinary literature regarding implant removal after fracture [13] and replacement [19]. However, all of these were reported in large dogs, whereas implants in small dogs were only reported in one study [20]. In this study, we report the successful replacement of a broken stem using the window technique after THR with the Zurich mini cementless total hip system in small dogs and cats.

## 2. Case Description

**Figure 1:**
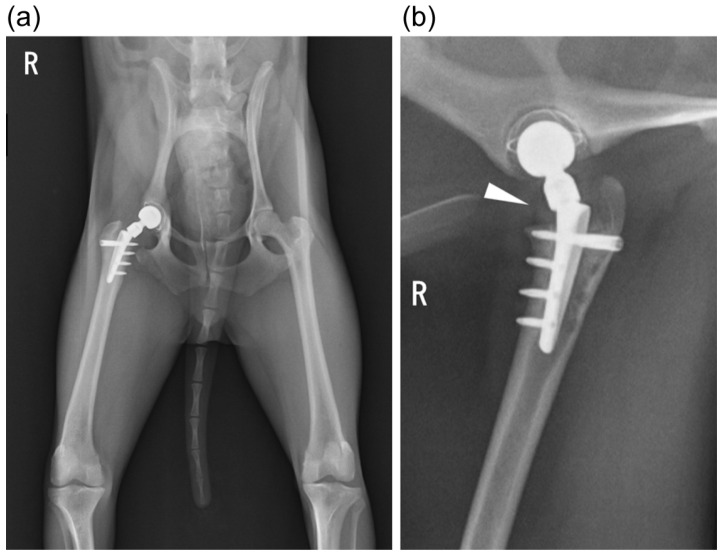
Postoperative radiographs after first total hip replacement surgery (right hip). (**a**) Ventrodorsal view; (**b**) yoga view: the stem was placed slightly proximally, as indicated by the arrowhead.

**Figure 2:**
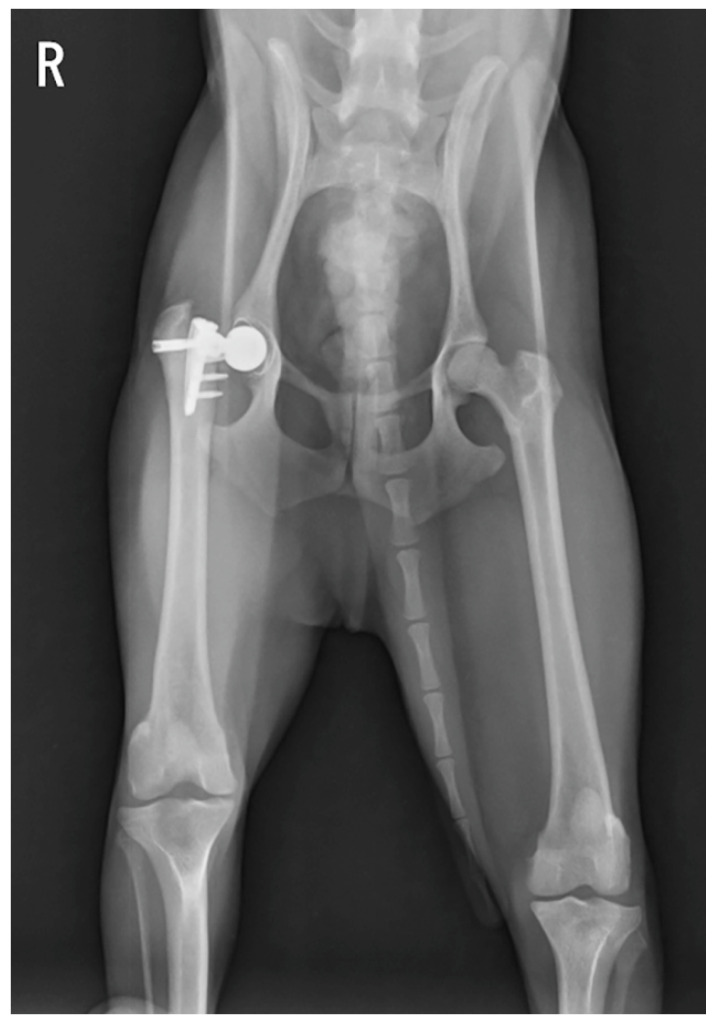
Ventrodorsal radiograph of the dog 176 days after the first surgery. This radiograph shows that the femoral stem fractured at the peg.

**Figure 3:**
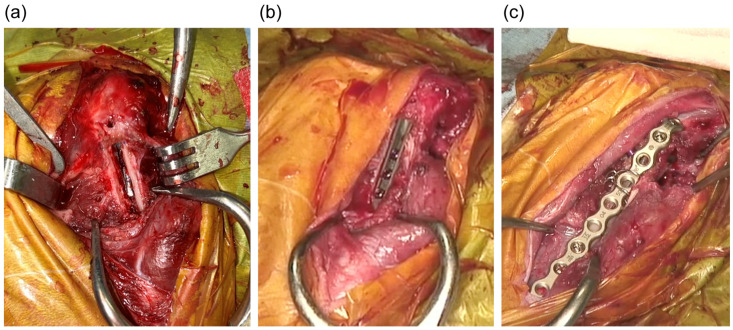
Intraoperative photo taken at revision surgery. (**a**) A window was created by cutting the lateral cortex of the femur using a high-speed bur and an oscillating saw; (**b**) a stem that is one size larger (SN; proximal width of 3.9 mm; distal width of 2.8 mm) fixed to the femoral medial cortex; (**c**) the bone fragments were put back in the window and a plate and a cerclage wire was applied over them.

The patient was a 2.8 kg, 1 year and 1 month old, neutered, male Toy Poodle. THR of the right hindlimb was performed for Legg–Calvé–Perthes disease. The Zurich mini-cementless total hip system was used for hip replacement. In this case, a size XS stem (3 mm wide) and size S neck made of Ti6Al7Nb titanium alloy were placed in the femur and an 11 mm cup of CFR-PEEK with a Ti/HA coating was placed in the acetabulum. The surgical procedure was performed as described in previous reports [20]. Postoperative radiographic examination revealed that the size and position of the implants were generally appropriate, except for the slightly proximal placement of the stem (Figure 1). The cup lateral opening, cup retroversion, and stem anteversion angles were 48°, 20°, and 23°, respectively. Lameness resolved 20 days postoperatively and rehabilitation was not required. Confinement in a restricted-space environment with non-slip flooring was required for 2 months. Leash-walking was allowed, while off-leash activity was not permitted (no running, jumping, or playing with other dogs). After clinical and radiographic re-examination 2 months after surgery, regular free activity was gradually permitted in the following month. Postoperative radiographs were taken at 2, 4, 8, and 12 weeks, followed by every 3 months until 1 postoperative year. On postoperative day 172, the patient presented with acute right-hind-limb lameness, and a radiographic examination was performed on postoperative day 176, which revealed a fracture of the peg portion of the stem (Figure 2). A second surgery was performed 1 month later. When the stem was reinstalled, general anesthesia was induced with propofol (4 mg/kg intravenously [IV]) and fentanyl (10 µg/kg IV) and maintained with isoflurane after endotracheal intubation. Analgesia was achieved through a constant-rate infusion of fentanyl (5–20 µg/kg/h). Ceftazidime (25 mg/kg IV) was administered 1 hour before the surgery and repeated every 2 h. After aseptic preparation, the approach was first performed from the anterolateral side of the femoral greater trochanter, and the joint was incised to confirm the fracture site. As the stem was firmly osseintegrated to the femur and impossible to pull out, a window was created using a high-speed bur and oscillating saw to separate the outer cortex into a rectangular shape approximately 3 mm wide, the same width as the stem, using a recently placed bicortical screw as a landmark to expose the outer part of the stem (Figure 3a). Since the stem was osseintegrated, it was removed via milling using a high-speed bur. This was performed with great care to avoid damage to the medial cortex of the femur.

**Figure 4:**
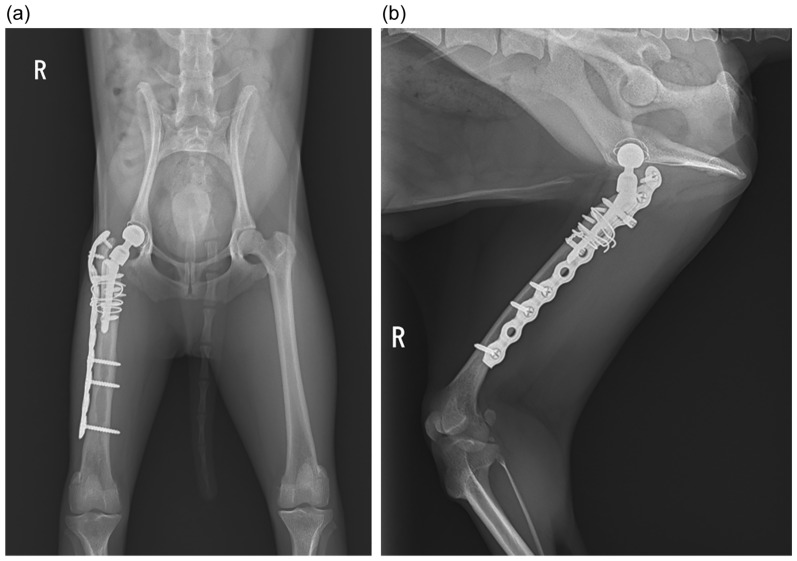
Postoperative radiographs 2 months after reimplantation: (**a**) ventrodorsal view; (**b**) yoga view (Cr-Cd view tangent to the stem).

After stem removal, the femoral canal was reamed with a rasp to allow for the insertion of the SN stem with a proximal width of 3.9 mm and distal width of 2.8 mm, which can accommodate a body weight of up to 6 kg. A new stem was then placed to avoid the screw hole from the first surgery (Figure 3b). The bone fragment was placed back in the window and temporarily fixed with a 22 G stainless steel wire. A titanium plate was then placed externally from the femoral greater trochanter to the femoral diaphysis, and a cerclage wire was made with a 22 G stainless steel wire to secure the detached bone fragments (Figure 3c). The gap between the created window and the bone fragments was filled using bone allograft (Fortigen-p; Progenica, Duleek, Ireland). The hip joint was then repositioned after a new stem was attached to a size S neck and an 8 mm head. The surgical field was cleaned, sutured, and closed. Bacterial cultures performed around the implants at the beginning and end of the second surgery were negative. Postoperative medical therapy consisted of ceftazidime (25 mg/kg IV twice daily), meloxicam (0.1 mg/kg SC once daily), and famotidine (1 mg/kg IV once daily) These medications were continued for 7 days postoperatively. The dog was discharged on the 7th postoperative day and oral cephalexin (26 mg/kg orally twice daily) was administered for 7 days after discharge. Postoperative radiographs were obtained at 2, 4, 8, and 12 weeks, and then every 3 months until 1 year postoperatively (Figure 4). The patient was able to bear their weight and walk postoperatively, and the lameness resolved after approximately a month; a rehabilitation program was not necessary. Approximately 1 year after surgery, no lameness was observed; additionally, no findings suggestive of pain or a limited range of motion upon palpation were observed, and the radiographic examination did not reveal any issues.

## 3. Discussion

The Zurich mini-cementless total hip system used in this study was introduced in 2020. Vezzoni et al. reported that 98% of small dogs and cats undergoing THR using a similar system obtained good outcomes. In the same report, complications were noted in seven of fifty cases, including stem breakage in two cases. In one case, the stem was removed, and in the other case, the stem was replaced. The patient who underwent replacement was followed up for 2 months postoperatively and reported no gait problems [20].

In humans, femoral stem fractures are generally considered to be caused by fatigue resulting from unfavorable biomechanics. The noted risk factors for stem fractures can be divided into three categories: patient, implant, and surgical factors. Notably, patient gender, body mass index, activity level, and bone loss in the proximal femur following revision THR were reported to increase the risk [21,22,23,24].

In this case, we observed breakage of the peg portion of the stem approximately 6 months after the first THR, and we replaced the stem successfully. The stem placed in the femur during the first THR surgery was XS in size, and the patient weighed 2.8 kg; the balance between the selected stem and the patient’s weight was adequate. However, in this case, the dog was very active, and excessive cyclical stress on the peg was considered the primary cause of the stem fracture. In addition, the stem was placed in a less-than-ideal proximal position, and in the first-generation XS stem, the transition between the peg and stem was at a right angle, which may have contributed to the stem breakage due to excessive stress.

Several window techniques have been reported in stem replacement surgery for femoral stem fractures in humans [25,26,27,28]. The only reports on femoral stem replacement using window techniques in dogs occurred in cases of cemented and cementless THR in large [19] and small dog breeds [20], respectively.

The postoperative course of the patients who underwent stem replacement surgery was relatively good. This suggests that the method of replacing the stem by creating a window on the lateral side of the femur and milling off the stem may be effective in small dogs in whom the stem cannot be removed after intimate osseointegration, as in the presented case.

However, when considering the details of the replacement surgery, it is most important to select a stem that is as large as possible according to the size of the femur and to carefully and meticulously cut the femoral lumen to place the stem at the optimal position and angle during the first surgery.

## 4. Conclusions

In conclusion, the use of the Zurich mini-cementless total hip replacement system in small dogs shows promising results, although complications such as stem fractures can occur. The use of the window technique for stem replacement proved to be an effective solution in this case, allowing for the successful removal and replacement of a fractured stem. This approach highlights the importance of careful stem selection and precise surgical techniques during initial THR to minimize the risk of complications. A limitation of this study, however, is that it is a follow-up report of a single case up to 1 year postoperatively. We believe that the usefulness of this technique should be further verified by increasing the number of cases and examining the long-term prognosis.

## Data Availability

The original contributions presented in the study are included in the article. Further inquiries can be directed to the corresponding author.

## Abbreviations

The following abbreviation is used in this manuscript:

THR	Total hip replacement
